# A Study on the Factors Influencing Triglyceride Levels among Adults in Northeast China

**DOI:** 10.1038/s41598-018-24230-4

**Published:** 2018-04-23

**Authors:** Anning Zhang, Yan Yao, Zhiqiang Xue, Xin Guo, Jing Dou, Yaogai Lv, Li Shen, Yaqin Yu, Lina Jin

**Affiliations:** Epidemiology and Biostatistics, School of Public Health, No. 1163 Xinmin Street, Jilin University, Changchun, Jilin, 130021 China

## Abstract

Triglyceride (TG) abnormalities are the most prevalent type of dyslipidaemia in the people of northeast China. Many researchers have investigated the prevalence, treatment and control of TG abnormalities, but little is known about the associations between the TG values and the factors that influence TG levels. This study aims to reveal quantile-specific associations of TG with its risk factors. A sample of 16,340 participants in Jilin Province were included in this study. A quantile regression (QR) model was performed to identify the factors that affected TG levels in different quantiles. The distribution of TG levels was different between males and females (*χ*^*2*^ = 155.77, *P* < 0.001). Body mass index (BMI) and waist circumference (WC) were positively associated with TG levels in all quantiles. Drinking was positively associated with TG levels in high quantiles (*P*_70.6_ to *P*_95_) only in males, while age had a positive association with TG levels in all quantiles only in females. The risk of WC on TG levels was higher with increasing TG levels, and smokers were more at risk for increasing TG levels, as well.

## Introduction

Dyslipidaemia is a lipid metabolic disorder that leads to a continuous increase in the plasmatic concentration of cholesterol and triglyceride (TG)^1^. It is believed to be a major cause of morbidity and mortality in most countries^2^ and is extremely burdensome on the health and economies of countries worldwide^3,4^. Hypertriglyceridaemia, a condition in which TG levels are elevated, is a common type of dyslipidaemia. In China, based on the Chinese Nutrition and Health Report in 2010, approximately 11.3% of Chinese adults require treatment for TG abnormalities with either lifestyle modification or the administration of medications^5–7^. Hypertriglyceridaemia is usually asymptomatic until triglycerides are greater than 1000–2000 mg/dl. Thus, although dyslipidaemia is believed to be a risk for coronary artery disease, hypertension and diabetes, it is easy to ignore.

Moderate exercise and drinking have shown to have a positive impact on TG levels^8^, whereas obesity, smoking, and poor eating habits have negative effects on TG levels^9,10^. However, TG was often a categorical variable in these studies, so some important information was ignored. On the one hand, continuous TG values can reflect the progression of dyslipidaemia to a certain extent because the occurrence and development of TG abnormalities is a continuous and long-term process. On the other hand, continuous TG values can also contribute to the progression of other diseases. For example, several epidemiologic studies have demonstrated that elevated TG levels were associated with increased cardiovascular disease (CVD) risk^11^ and the occurrence and development of pancreatitis, stroke and diabetic microvascular complications^12^. Therefore, investigating the associations between TG values and influencing factors is of great importance. It is also a challenge to examine the different factors that influence different TG levels rather than examining the factors that influence the mean TG levels.

Ordinary least squares (OLS) regression was one of the most commonly used statistical methods in previous studies; however, OLS cannot fully reflect the whole distribution^13^ because it focuses on the average. In addition, OLS is not suitable for modelling data with heterogeneous conditional distributions, which would lead to an estimation bias^14^. Quantile regression (QR)^15^ is a direct extension of OLS regression, which could analyse the margin effect of each specific quantile condition^16^, and the least absolute value (WLAV) method was used to estimate parameters^17^. Further, QR has great flexibility in modelling data with heterogeneous conditional distributions without distributional assumption in the model^17,18^. Thus, QR models were used to identify the associations between influencing factors and TG (especially the low and high quantiles of TG).

In this study, the associations between the values of TG and the influencing factors were investigated using QR models. Participants were part of the study on the prevalence of chronic diseases and risk factors among adults in Jilin Province, which is located in the central part of northeast China and has a temperate continental monsoon climate^19^. The QR model was performed to provide a series of coefficients in different quantiles of TG levels to find specific associations between the factors and TG levels.

## Results

### Descriptive characteristics of participants by gender

As shown in Table 1, the BMI, WC and FBG were significantly higher in males than in females (*P* < 0.05). The distribution of TG levels was different between males and females (*χ*^*2*^ = 155.77, *P* < 0.001). The proportions of demographics (occupations, educational level, smoking, drinking, hypertension and diabetes) were significantly different between the two genders (*P* < 0.05). In addition, there were significant differences between males and females (*P* < 0.05) in their dietary habits and the mode of transportation.

**Table 1:** Descriptive characteristics of participants by gender [$\overline{X}$ ± SD, n(%)]

Variables	Total (n = 16340)	Male (n = 7528)	Female (n = 8812)	*t*/*χ*^2^	*P* value
Age (year)^*^	47.40 ± 13.26	46.76 ± 13.92	47.94 ± 12.65	−5.71	<0.001
BMI (kg/m^2^)^*^	24.12 ± 3.68	24.19 ± 3.68	24.05 ± 3.68	2.26	0.024
WC (cm)^*^	81.99 ± 10.54	84.28 ± 10.43	80.03 ± 10.24	26.10	<0.001
Distribution of TG^*^				155.77	<0.001
Normal triglyceride	9828(60.2)	4206(55.9)	5622(63.8)		
Borderline high	2442(14.9)	1109(14.7)	1333(15.1)		
Hypertriglyceridaemia	4070(24.9)	2213(29.4)	1857(21.1)		
Residence				3.43	0.064
Rural	8120(50.3)	3682(48.9)	4438(50.4)		
Urban	8220(49.7)	3846(51.1)	4374(49.6)		
Occupations^*^				476.89	<0.001
Others	3799(23.2)	1165(15.5)	2634(29.9)		
Mental labour	3064(18.8)	1506(20.0)	1558(17.7)		
Manual labour	9477(58.0)	4857(64.5)	4620(52.4)		
Educational level^*^				191.44	<0.001
Compulsory	9753(59.7)	4061(53.9)	5692(64.6)		
High school	4148(25.4)	2190(29.1)	1958(22.2)		
Undergraduate	2439(14.9)	1277(17.0)	1162(13.2)		
Smoking^*^				3207.61	<0.001
No	11214(68.6)	3492(46.4)	7722(87.6)		
Yes	5126(31.4)	4036(53.6)	1090(12.4)		
Drinking^*^				4198.82	<0.001
	No	11243(68.8)	3267(43.4)	7976(90.5)	
	Yes	5097(31.2)	4261(56.6)	836(9.5)	
Hypertension^*^				94.96	<0.001
No	10568(64.7)	4572(60.7)	5996(68.0)		
Yes	5772(35.3)	2956(39.3)	2816(32.0)		
Diabetes^*^				5.26	0.022
No	14906(91.2)	6826(90.7)	8080(91.7)		
Yes	1434(8.8)	702(9.3)	732(8.3)		
Vegetable^*^				70.07	<0.001
Occasionally/rarely	1686(10.3)	939(12.5)	747(8.5)		
Often	14654(89.7)	6589(87.5)	8065(91.5)		
Fruit^*^				282.80	<0.001
Occasionally/rarely	7995(48.9)	4219(56.0)	3776(42.9)		
Often	8345(51.1)	3309(44.0)	5036(57.1)		
Meat^*^				531.75	<0.001
Occasionally/rarely	10759(65.8)	4260(56.6)	6499(73.8)		
Often	5581(34.2)	3268(43.4)	2313(26.2)		
Fish^*^				118.06	<0.001
Occasionally/rarely	14676(89.8)	6552(87.0)	8124(92.2)		
Often	1664(10.2)	976(13.0)	688(7.8)		
Eggs/Bean/Bean products^*^				38.30	<0.001
Occasionally/rarely	6514(39.9)	2808(37.3)	3706(42.1)		
Often	9826(60.1)	4720(62.7)	5106(57.9)		
Mike/Dairy products^*^				10.91	<0.001
Occasionally/rarely	13869(84.9)	6465(85.9)	7404(84.0)		
Often	2471(15.1)	1063(14.1)	1408(16.0)		
Mode of transportation^*^				241.70	<0.001
Walking/bicycling	9005(55.1)	3656(48.6)	5349(60.7)		
Driving/riding	7335(44.9)	3872(51.4)	3463(39.3)		

$\bar{x}$: mean; SD: standard deviations; n: number.

^*^P < 0.05, “other” included unemployed and retired people

BMI: body mass index; WC: waist circumference; TG: triglyceride.

### The distribution of TG between males and females

**Figure 1:**
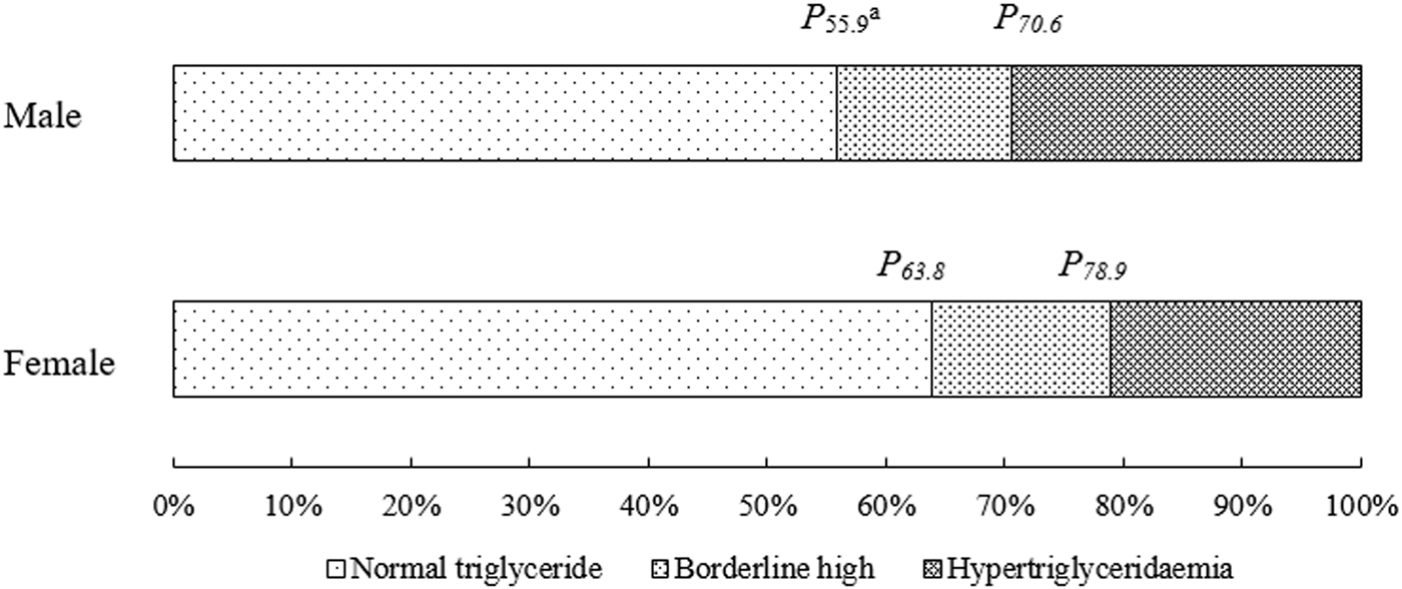
The distribution of TG between males and females (^a^*P*_x_ was used to represent the percentile x).

Figure 1 and Supplementary Table 1 show the distribution of TG levels along with the corresponding quantiles of the critical values. The differences between males and females were obvious and statistically significant, so the QR model was performed separately by gender afterwards.

### QR model performance

Tables 2 and 3 list the coefficients of multivariate QR between the TG levels and their influencing factors for males and females independently. In males, WC and hypertension were positively associated with TG levels in all quantiles, and the coefficients of WC and hypertension showed an increasing trend with TG levels. BMI was positively association with TG levels in all quantiles and displayed an increasing trend, especially in the middle and high quantiles (*P*_60.5_ to *P*_95_). Furthermore, smoking status was positively correlated with TG levels compared with non-smokers, and the regression coefficients had a significant increasing tendency in high quantiles (*P*_70.6_ to *P*_95_). Drinking status and diabetes had similar effects. Compared with people who always walked/biked, those who drove/rode cars had higher risk for increasing TG levels in the middle and high quantiles (*P*_60.5_ to *P*_95_).

**Table 2:** Quantile regression coefficients between TG and variables for males.

Factors	Normal triglyceride						Borderline high				Hypertriglyceridaemia					
	*P*_10.0_		*P*_25.0_		*P*_50.0_		*P*_55.9_		*P*_60.5_		*P*_70.6_		*P*_75.4_		*P*_95.0_	
	*β*	*P*	*β*	*P*	*β*	*P*	*β*	*P*	*β*	*P*	*β*	*P*	*β*	*P*	*β*	*P*
Smoking	0.083	<0.001	0.123	<0.001	0.172	<0.001	0.197	<0.001	0.204	<0.001	0.240	<0.001	0.263	<0.001	0.658	<0.001
Drinking	−0.023	0.140	0.044	0.008	0.126	<0.001	0.151	<0.001	0.178	<0.001	0.168	<0.001	0.255	<0.001	1.083	<0.001
Mode of transportation	0.048	0.002	0.067	<0.001	0.099	<0.001	0.115	<0.001	0.122	<0.001	0.123	<0.001	0.122	0.003	0.380	0.009
Diabetes	0.117	<0.001	0.178	<0.001	0.344	<0.001	0.435	<0.001	0.513	<0.001	0.622	<0.001	0.696	<0.001	3.723	<0.001
Hypertension	0.069	<0.001	0.089	<0.001	0.127	<0.001	0.134	<0.001	0.151	<0.001	0.210	<0.001	0.235	<0.001	0.495	0.002
BMI	0.021	<0.001	0.031	<0.001	0.046	<0.001	0.057	<0.001	0.060	<0.001	0.081	<0.001	0.095	<0.001	0.155	<0.001
WC	0.010	<0.001	0.016	<0.001	0.027	<0.001	0.028	<0.001	0.030	<0.001	0.034	<0.001	0.035	<0.001	0.069	<0.001

BMI: body mass index; WC: waist circumference; Hypertriglyceridaemia: TG ≥ 2.26 mmol/L; Borderline high: TG < 2.26 mmol/L and TG ≥ 1.7 mmol/L; Normal triglyceride: TG<1.7 mmol/ L.

**Table 3:** Quantile regression coefficients between TG and variables for females.

Factors	Normal triglyceride						Borderline high				Hypertriglyceridaemia					
	*P*_10.0_		*P*_25.0_		*P*_60.5_		*P*_63.8_		*P*_75.4_		*P*_78.9_		*P*_90.0_		*P*_95.0_	
	*β*	*P*	*β*	*P*	*β*	*P*	*β*	*P*	*β*	*P*	*β*	*P*	*β*	*P*	*β*	*P*
Urban	0.010	0.395	0.043	0.002	0.071	0.002	0.082	<0.001	0.107	<0.001	0.125	<0.001	0.149	0.006	0.171	0.099
Age	0.006	<0.001	0.008	<0.001	0.013	<0.001	0.013	<0.001	0.015	<0.001	0.016	<0.001	0.017	<0.001	0.020	<0.001
Smoking	0.090	<0.001	0.096	<0.001	0.185	<0.001	0.180	<0.001	0.253	<0.001	0.232	<0.001	0.462	<0.001	0.584	0.023
Diabetes	0.159	<0.001	0.227	<0.001	0.592	<0.001	0.665	<0.001	0.872	<0.001	1.001	<0.001	1.346	<0.001	1.947	<0.001
Hypertension	0.075	<0.001	0.095	<0.001	0.160	<0.001	0.161	<0.001	0.192	<0.001	0.203	<0.001	0.407	<0.001	0.518	0.002
BMI	0.007	0.011	0.013	<0.001	0.018	<0.001	0.017	0.001	0.024	0.002	0.024	0.004	0.039	0.008	0.075	0.008
WC	0.007	<0.001	0.011	<0.001	0.020	<0.001	0.022	<0.001	0.028	<0.001	0.030	<0.001	0.041	<0.001	0.045	<0.001

BMI: body mass index; WC: waist circumference; Hypertriglyceridaemia: TG ≥ 2.26 mmol/L; Borderline high: TG<2.26 mmol/L and TG ≥ 1.7 mmol/L; Normal triglyceride: TG< 1.7 mmol/ L.

Similarly, WC, smoking, hypertension and diabetes in females had similar effects as those in males (Table 3). BMI also showed a positive association with TG levels in all quantiles and displayed a slightly increasing trend in the low and middle quantiles (*P*_10_ to *P*_75.4_), and the regression coefficients had a significant increasing tendency in the high quantiles (*P*_78.9_ to *P*_95_). Age had a positive association with TG levels in all quantiles in females, and females who lived in rural areas were positively associated with increasing TG levels (*P*_10_ to *P*_95_), compared with those who lived in urban areas.

## Discussion

In many studies, TG was treated as a categorical variable, or the mean TG was used. Thus, although influencing factors for TG abnormalities have been extensively explored, little is known about associations between the factors and different levels of TG. In our study, TG was treated as a continuous variable, and the QR model was performed to explore the associations between factors and the progress of TG elevations.

Abdominal obesity has been well established to be linked to dyslipidaemia^20^. Obesity was believed to decrease the activity of lipolysis through insulin resistance, eventually leading to the failure of TG clearance^21,22^. In our study, BMI was positively associated with TG in all quantiles. However, the trends in the relationships between the BMI and TG were different in males and females. In males, as TG levels increased, the association was stronger, especially in high quantiles of TG levels (such as *P*_60.5_ to *P*_95_). By contrast, in females, the trend was different. Although BMI and TG were always positively associated with each other in females, the BMI was strongly and positively associated with TG levels only in high quantiles. This finding suggests that as TG increases to high levels, it is more sensitive to the increase in BMI. This finding indicated that control of one’s BMI should be more emphasized and strengthened among those with high levels of TG in females, while controlling the BMI should always be on the table in males as TG increases. This important information cannot be found in the traditional model when TG was treated as a categorical variable.

Generally, smoking was a risk factor for dyslipidaemia, especially in those with elevated TG levels^23,24^. In our study, smoking was positively associated with TG levels compared with those who did not smoke. Meanwhile, studies showed that females were at greater risk from smoking than males^10^. Therefore, smoking cessation is more necessary in females. Furthermore, drinking was shown to be a predictor for higher TG levels in males, with a larger extent of the positive association at a higher quantile. This finding was consistent with the findings in the literature that non-drinkers usually had a lower risk for elevated TG levels^9^. However, there was no evidence that the intake of alcohol could affect TG levels in females, a feature that may be attributed to fewer female drinkers^25^. In addition, compared with males who always walk or bike, those who drove or rode cars had a higher risk for TG abnormalities. The association was stronger, especially in the high quantiles of TG levels (such as *P*_60.5_ to *P*_95_). This finding suggests that, as TG increase to high levels, it will be more sensitive to the mode of transportation. Furthermore, studies have shown that moderate exercise is beneficial for maintaining normal and stable TG levels^26^. Thus, males with high levels of TG are recommended to change sedentary habits and engage in regular exercise more frequently.

Diabetes is clearly associated with dyslipidaemia^27^. Diabetes is a chronic metabolic disease with high blood glucose as the main biochemical characteristic, while those with diabetes are more likely already to have had a lipid metabolism disorder. Our results have confirmed this finding. The mechanism may be related to insulin resistance and insulin sensitivity^28^. Meanwhile, the elevated TG levels could also cause higher free fatty acid levels in the serum, which would lead to a reduction in islet β-cell function^29^. Similarly, studies have shown that high blood pressure were associated with lipid metabolism disorders, and plasma TG levels were significantly higher than normal^30^. Patients with hypertension would attain more risk in TG.

Some limitations should be noted. First, the data were derived from a cross-sectional study in Jilin Province, which may limit the generalizability of our results to other regions. Second, health-related behaviours and dietary habits were reported by the participants themselves, which may lead to a reporting bias. Third, some of the variables were not quantified. Thus, more details were neglected, e.g., the amount of the alcohol consumption. Fourth, several potential confounders, such as glucose metabolism and genes, were not under consideration in this study, which may have slightly affected our results.

## Conclusion

The risk of WC for TG was positive, and the risk is higher with increasing TG levels. Smokers and those with diabetes or hypertension are at increased risk for TG abnormalities, as well. Males with normal or borderline high TG levels are at high risk for the increase in BMI and alcohol consumption. Meanwhile, the risk of elevated TG is expected to increase with age in females. This finding suggests that middle-aged and elderly females should pay more attention to their TG levels.

## Methods

### Study population

**Figure 2:**
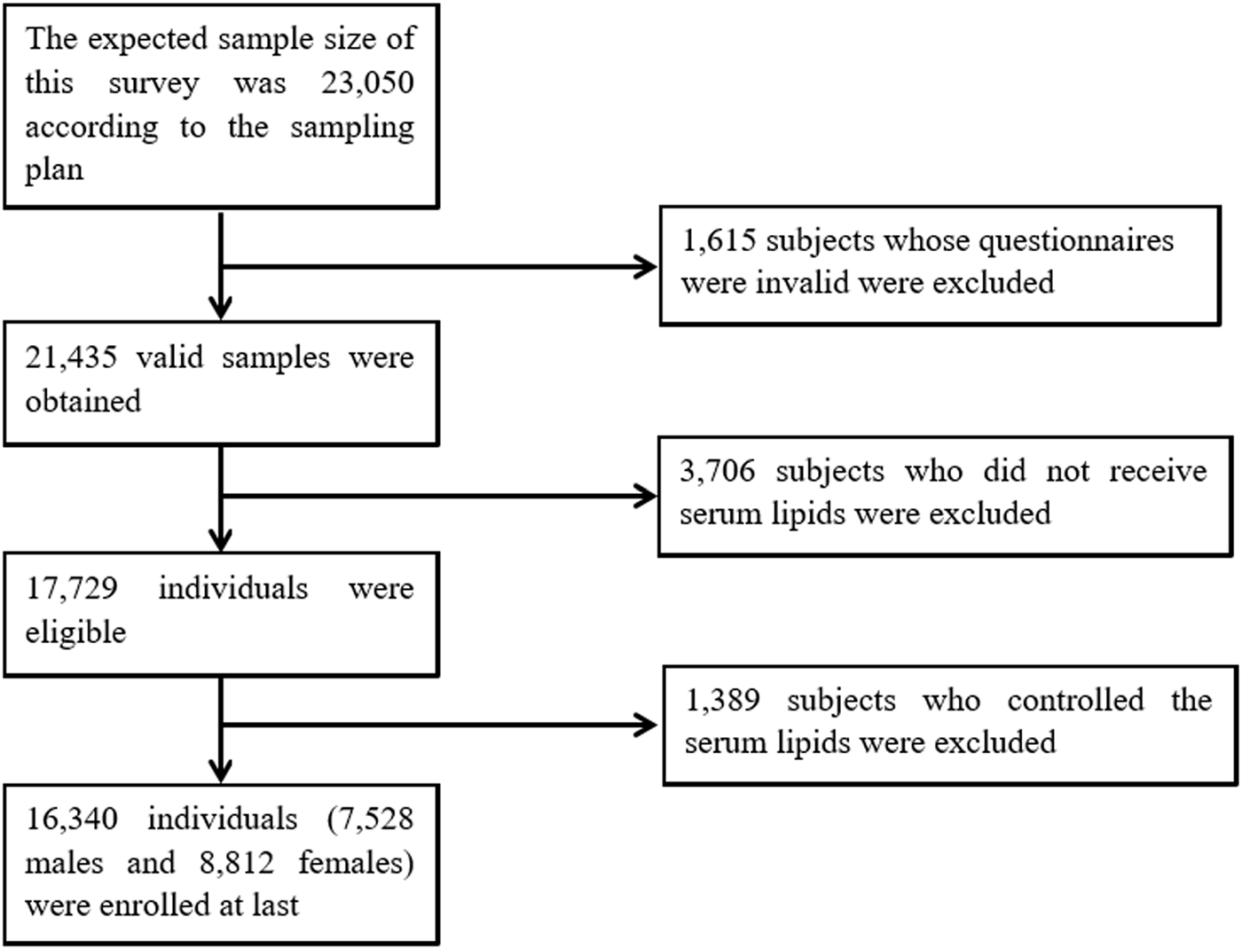
Study design and participant flow diagram for the present study.

The data were derived from a cross-sectional study of adult chronic disease, and the risk factors in Jilin Province, China, in 2012 were measured by the School of Public Health, Jilin University, and the Jilin Department of Health. Multistage stratified random cluster sampling was used to select a total of 23,050 participants aged 18–79 years old who had lived in Jilin Province for more than 6 months^31^ (see details in Part 1 of the Supplementary Material online). This study investigated the association between TG levels and related risk factors. For this purpose, some subjects were excluded due to those who did not get tested (3,706 subjects) or had serum lipid control (1,389 subjects). The ultimate target sample size included a total of 16,340 people in this study (Fig. 2). All of the participants provided written informed consent, and the study was approved by the Institutional Review Board of the School of Public Health, Jilin University. All of the methods were performed in accordance with the relevant guidelines and regulations.

### Data Collection and Measurement

To obtain informed consent, all of the participants were interviewed face-to-face by unified and trained investigators. Data, including demographic information (e.g., gender, age, occupation), behavioural factors (e.g., smoking, drinking, physical activity), dietary habits (e.g., vegetables, fruits, meats, eggs), chronic disease prevalence within one year (e.g., hypertension, diabetes) and anthropometric measurements (e.g., height, weight, TG levels) were taken. The participants’ height, weight and waist circumference (WC) were measured using a standardized protocol and process, with participants wearing clothing but no shoes. Blood glucose was monitored using the Bayer Bai Ankang fingertip blood glucose monitor machine (Bayer, Leverkusen, Germany) and test paper. Serum lipids were measured using the MODULE P800 biochemical analyser and original auxiliary reagents the morning after participants fasted for more than 10 hours overnight.

### Assessment Criteria

Hypertriglyceridaemia was defined as TG ≥ 2.26 mmol/L. Borderline high was defined as TG < 2.26 mmol/L and TG ≥ 1.7 mmol/L. Normal TG was defined as TG < 1.7 mmol/ L^32^. Hypertension was defined as a resting systolic blood pressure (SBP) ≥ 140 mmHg and/or a diastolic blood pressure (DBP) ≥ 90 mmHg and/or by the use of antihypertensive medications in the past two weeks^33^. Diabetes mellitus (DM) was defined as the use of hypoglycaemic agents or a self-reported history of diabetes or fasting blood glucose (FBG) ≥ 7.0 mmol /L^34^. Smoking was defined as a person who smoked more than daily over the past 30 days. Drinking was defined as a person who consumed an average of more than one alcoholic drink per week, whether the drink was in the form of spirits, beer, wine or other forms of alcohol. Physical labour included production workers, farmers and service workers. Mental labour included office and other technical personnel. Other occupations included students, the unemployed and retirees. Body mass index (BMI) was calculated as the weight (kg)/height (m) ^2^. Dietary habits were classified into two types: “Occasionally/rarely” and “Often”. “Occasionally/rarely” was defined as a person who eats less than three times per week. “Often” was defined as a person who eats three times per week or more. The mode of transportation was divided into two types: “Walking/bicycling” and “Driving/riding”.

### Statistical Analysis

The continuous variables were described by using the means ± standard deviations (SD) or the rate and was compared using Student’s t-test. While the categorical variables were described as counts or percentages and were compared using the Rao-Scott Chi-square test. The QR model was used to identify the influencing factors in different quantiles of TG measurements. We defined the candidate variables to be included in the analysis were that could influence the TG levels after referred to a large number of relevant literature and combined the data we have collected and measured. (1) Table 1 lists all of the candidate factors involved in the QR analysis. (2) The multivariate QR analysis was conducted, and (3) the backward stepwise regression was used to select variables. Only the significant variables were retained in the final model and listed in Tables 2 and 3. Statistical analyses were carried out using the R version 3.3.2 (University of Auckland, Oakland, New Zealand). *P* < 0.05 was considered statistically significant.

### Data availability

The survey was implemented by the School of Public Health at Jilin University and the Jilin Center for Disease Control and Prevention in Jilin Province in 2012. Unfortunately, due to relevant regulations, the data cannot be shared.

## Electronic supplementary material

Supplementary Material
