# Supplementary material for: A Study on the Factors Influencing Triglyceride Levels among Adults in Northeast China
Source: Sci Rep. 2018 Apr 23;8:6388. doi: 10.1038/s41598-018-24230-4 (PMC5913297; doi:10.1038/s41598-018-24230-4)
Supplement: Supplementary file 1 — Supplementary Material [file 41598_2018_24230_MOESM1_ESM.pdf]

Online Supplementary Material to

A Study on the Factors Influencing Triglyceride Levels among Adults in Northeast China

Anning Zhang[a], Yan Yao[a,*], Zhiqiang Xue[a], Xin Guo[a], Jing Dou[a], Yaogai Lv[a], Li Shen[a], Yaqin Yu[a], Lina Jin[a,*]

[a] Epidemiology and Biostatistics, School of Public Health, No.1163 Xinmin Street, Jilin University, Changchun, Jilin, China, 130021

* Correspondence: jinln@jlu.edu.cn, Tel.: +86-431-85619451;

yaoyan@jlu.edu.cn; Tel.: +86-431-85619456;

1. Sampling Method

Five-stage stratified random cluster sampling was used to select the samples under study. In the first stage, 32 districts/counties were identified in proportion to population, geographic location and ethnicity, from nine cities (Changchun, Jilin, Siping, Liaoyuan, Tonghua, Baishan, Songyuan, Baicheng and Yanbian). At the second stage, three or four towns (depending on the size of the district) were selected by stratified random sampling to guarantee the representativeness of each sample. In the third stage, three neighborhood committees were chosen by stratified random sampling from each of the towns previously selected. In the fourth stage, one village from each chosen neighborhood committee was selected by simple random sampling. In the final stage, cluster random sampling was used to identify individuals aged 18 to 79 years old from each of the villages selected for the study.
